# Feasibility of a peer-led, after-school physical activity intervention for disadvantaged adolescent females during the COVID-19 pandemic: results from the Girls Active Project (GAP)

**DOI:** 10.1186/s40814-022-01149-2

**Published:** 2022-08-30

**Authors:** Sara McQuinn, Sarahjane Belton, Anthony Staines, Mary Rose Sweeney

**Affiliations:** 1 School of Nursing, Psychotherapy and Community Health, Faculty of Science and Health, Dublin City University, Dublin 9, Ireland; 2 School of Health and Human Performance, Faculty of Science and Health, Dublin City University, Glasnevin, Dublin 9, Ireland

**Keywords:** Physical activity, Adolescents, Female, Behaviour change, Feasibility, Intervention, Peer led, School, Mixed methods, Implementation, COVID-19

## Abstract

**Introduction:**

There is a critical need for interventions that can be feasibly implemented and are effective in successfully engaging adolescent females in physical activity (PA). A theory-based, peer-led, after-school PA intervention, the *Girls Active Project* (GAP), was codesigned with adolescent females. This study aimed to assess the feasibility of implementing and evaluating the GAP programme.

**Setting:**

One single-sex, female-only, designated disadvantaged postprimary school (students aged 12–18) in Dublin, Ireland.

**Methods:**

Mixed methods were applied with multiple stakeholders over a 12-week trial (March to May 2021). A single-arm study design was used to examine intervention: reach, dose, fidelity, acceptability, compatibility and context. Feasibility of using proposed self-reported outcome measures (moderate-to-vigorous PA levels, self-rated health, life satisfaction, PA self-efficacy and PA enjoyment) was also explored. Due to school closure resulting from the COVID-19 pandemic, the intervention was delivered both online and in person in the school setting.

**Results:**

Eight exercise classes were peer delivered by project leaders (*n* = 6, students aged 15–17) to intervention recipients (students aged 13-14). Recruitment was low (*n* = 8, 10% of eligible students, mean age: 13.3 *SD*: 0.46), yet retention was high (*n* = 7/8, 88%). Attendance rates were satisfactory (68%), and the intervention was implemented with high fidelity (87%). Data completion rates suggested proposed self-reported outcome measures were deemed appropriate (≥ 95%), except for weight (50%) and height data (80%). Despite COVID-19 hindering intervention implementation, both quantitative and qualitative data suggested that stakeholders were satisfied and perceived the in-person delivered intervention to be compatible with the school setting. Recommended refinements included extending class duration, introducing different rewards, and boosting programme awareness.

**Conclusions:**

Further thought must be given on how to increase recruitment. Overall, the in-person delivered after-school PA programme was well-received by stakeholders and shows promise as an intervention that can be feasibly implemented and evaluated. Suggested improvements to the GAP intervention programme are recommended, before continuing to a more robust evaluation.

**Trial registration:**

10.17605/OSF.IO/75HWJ (prospectively registered, date of registration: 9th December 2020)

**Supplementary Information:**

The online version contains supplementary material available at 10.1186/s40814-022-01149-2.

## Key messages regarding feasibility

- What uncertainties existed regarding the feasibility? It was unclear if the Girls Active Project programme to increase adolescent females PA levels could be successfully implemented and evaluated in a postprimary school setting.
- What are the key feasibility findings? Recruitment was low, yet retention was high. The intervention was implemented as intended, and most of the proposed self-reported outcome measures were deemed appropriate. Stakeholders indicated satisfaction with the in-person delivered intervention and perceived it to be compatible with the school setting.
- What are the implications of the feasibility findings for the design of the main study? The in-person delivered intervention shows promise; however, further work is required to revise and test recruitment strategies. Prior to the main study, refinements to the intervention should be made based on stakeholder recommendations, while further consideration needs to be given to retaining proposed self-reported outcome measures weight and height,  and introducing direct observation to assess fidelity.

## Background

Despite evidence continuing to accumulate on the health benefits of regular physical activity (PA) for adolescents [1, 2], globally, 85% of adolescent females (aged 11–17) are insufficiently physically active [3]. PA levels are particularly low among females of lower socio-economic status [4, 5]. The World Health Organization recommends that adolescents accumulate at least an average of 60 min a day of moderate-to-vigorous intensity physical activity (MVPA) [6]. An age-related decline in PA participation during adolescence is a consistent finding in the literature [7, 8], with national evidence to suggest [9] that the largest reduction in PA levels for females occurs between second (aged 13–14) and third year (aged 14–15) in postprimary school. Efforts made to increase the PA levels of adolescents are of particular importance since PA appears to track reasonably well from adolescence to adulthood [10]. Furthermore, there is evidence emerging to suggest that the on-going COVID-19 pandemic has caused a further decrease in PA participation [11, 12], especially for adolescents of lower socio-economic status [11].

There is a critical need to strengthen the development and implementation of effective interventions to increase adolescent female PA levels [3, 13]. The school environment is well-known as a potential setting for targeting adolescent PA behaviour [14]. Evidence suggests, however, school-based PA interventions have been minimally successful at increasing PA levels [15–18]. This indicates that changing adolescent PA behaviour through school-based interventions can be challenging. Previous reviews suggest that multicomponent [16, 19] interventions and interventions that use a theoretical model or framework [18, 19] might be most effective in the promotion of PA for adolescents, with certain intervention strategies, such as after-school PA programmes conducted in the school setting showing potential [20].

At postprimary school level in Ireland, traditional team-based and structured sports dominate extra-curricular PA, i.e., PA or sport played before, during or after school, but not part of the curriculum [21]. Given that participation is higher among males (70%) than females (57%) and in more affluent students (66%) than less affluent students (56%) [21], a call for additional extracurricular PA programmes that appeal to adolescent females of lower socio-economic status may be warranted. In Ireland, a classification system known as DEIS (Delivering Equality of opportunity in Schools) is used by the Department of Education to indicate that a school is based in a community at risk of disadvantage and social exclusion [22]. The Girls Active Project (GAP) programme was codesigned with adolescent females in a designated disadvantaged (DEIS) postprimary school in Ireland using the behaviour change wheel [23] in combination with a public and patient involvement (PPI) approach. This novel approach was applied to systematically codesign a contextually appropriate school-based and theory-based PA intervention that aimed to meet the needs of adolescent females and provide relevant and meaningful opportunities for them to be active. Details of the intervention development process are available elsewhere [24]. The GAP is a novel, multicomponent, peer-led PA programme. It offers female adolescents a readily accessible fun and unstructured opportunity to be active with peers in a supportive and inclusive environment. The females who codesigned the GAP programme chose for the programme to be peer delivered after school in the school setting. The GAP includes strategies that are commonly recommended across the literature to promote adolescent females’ PA, such as incorporating social support [25, 26] and providing females with autonomy through choice of activity, alongside offering a diverse range of activities [27–29]. Moreover, given that lack of time has been identified as a key barrier to school PA policy implementation [30], engaging students to deliver intervention components may possibly reduce the burden on teachers [31]. Using a peer-led approach (such as that used in the GAP programme) in school-based interventions has become increasingly popular and has shown potential in increasing adolescent female PA levels [32–34], with findings to suggest that peer-led PA interventions may be equally as effective as those delivered by professionals [35]. The next step was to assess feasibility of the GAP intervention programme.

There is a growing appreciation of the significant role that feasibility studies play in the development and evaluation of complex interventions, primarily its value of progressing to a larger-scale trial or effectiveness study [36–38]. This small-scale real-world testing can provide information for researchers to enhance the thoroughness of a future trial [39, 40] and through publication may benefit other researchers [41, 42]. Following the Medical Research Council framework guidance [36], this study aimed to investigate the feasibility of implementing and evaluate the GAP intervention (Trial number: 10.17605/OSF.IO/75HWJ). The research team selected the feasibility measures deemed most appropriate for this study based on the research objectives and available data [43]. These included the following: reach, dose, fidelity, acceptability, compatibility and context. This study also explored the feasibility of measuring the proposed self-reported outcomes (minutes of daily moderate-to-vigorous PA (MVPA), height, weight, self-rated health, life satisfaction, self-efficacy related to PA and PA enjoyment) that will be used to evaluate efficacy in a future trial [41], by assessing their completion rates. Reporting data completion rates is consistent with previous feasibility studies [44, 45] and proves useful as this information can help inform intervention refinements and provide additional support for the feasibility of using these outcome measures [43].

The specific objectives of this feasibility study were as follows:

1. Capture the recruitment and retention rates of intervention recipients and explore factors influencing participation (reach).
2. Determine attendance rates and the extent to which intervention providers implemented the intervention as intended (dose and fidelity).
3. Assess the feasibility of using proposed self-reported outcome measures (data completion rates).
4. Explore stakeholders’ satisfaction with the intervention (acceptability).
5. Examine the perceived fit and sustainability of the intervention in the school setting (compatibility).
6. Understand context, i.e. the external factors that affected intervention implementation (context).

## Method

### Design and setting

A mixed-methods single-arm feasibility trial was conducted in a female-only, designated disadvantaged postprimary school in Dublin, Ireland. This school had previously participated in the codesign of the GAP programme [24] and was therefore familiar with the intervention and proposed processes involved. To ensure it reflected the realities of the intervention setting [37], this study involved multiple stakeholders (intervention recipients, intervention providers, school staff and parents/guardians). The feasibility measures and their definitions, stakeholders involved, data collection tools and the timeframe of each objective as they relate to the present study are provided in Table 1. The reporting of this study followed the Consolidated Standards of Reporting Trials (CONSORT) 2010 [46] statement with extension to randomised pilot and feasibility trials (Supplementary file 1) and the Template for Intervention Description and Replication (TIDieR) checklist [47] (Supplementary file 2). Ethical approval for this study was granted by the Dublin City University Research Ethics Committee (DCUREC/2019/005).

**Table 1:** Data collection procedures and schedule of measures

Objective	Measure ***Definition***	Stakeholder(s)	Data collection tool	Data collection timeframe				
				Week 1: phase 1 baseline	Week 8: mid	Week 8: phase 2 baseline	Week 12: post	During (throughout week 1-12)
				Online	Paper based/in person	Paper based/in person	Online/paper based/in person	Online/in person
1. To capture the recruitment and retention rates of intervention recipients and explore factors influencing participation	**Reach**^**a**^ *Participation rate in the innovation by the intended audience*	Intervention recipients	Recruitment and retention records (i.e. #who were eligible, #who consented, #who enrolled, #who stayed)	X	X	X	X	X
			Demographic section in questionnaire	X		X^c^		
			Feedback questionnaire and focus group		X		X	
		Parents/guardians	Questionnaire				X	
2. To determine attendance rates and the extent to which intervention providers implemented the intervention as intended	**Dose**^**b**^ ***Dose delivered:*** *The number/amount of intended units delivered/provided (i.e. dose is a function of the efforts of intervention providers)* ***Dose received****: Extent to which participants engage or interact with is receptive or use intervention (i.e. dose is a function of the efforts of intervention participants)* **Fidelity**^**b**^ *The extent to which the programme was implemented as planned*	Intervention recipients	Attendance records					X
		Intervention providers	Project leader logbooks					X
3. To assess the feasibility of using proposed self-reported outcome measures	‘Data completion rates’ of outcome measures were used as an indicator to the following: **Feasibility of future trial design to conduct a full trial**^**a**^ *Measures informing implementation trial methods including the feasibility, acceptability, or quality of data collection procedures, survey items, tools, or data management strategies*	Intervention recipients	#Outcome measures completed (% data completion)	X	X	X^c^	X	
4. To explore stakeholders’ satisfaction with the intervention	**Acceptability**^**a**^ *Service providers or support system’s satisfaction with the innovation*	Intervention recipients	Feedback questionnaire and focus group		X		X	
		Intervention providers	Feedback questionnaire				X	
		Intervention providers	Focus group		X		X	
		Parents/guardians	Questionnaire and semi-structured interview				X	
		School staff	Semi-structured interview				X	
5. To examine the perceived fit and sustainability of the intervention in the school setting	**Compatibility (appropriateness**)^a^ *Perceived fit of the innovation with organisation’s values, mission ,and priorities*	Intervention recipients	Feedback questionnaire and focus group		X		X	
		Intervention providers	Feedback questionnaire				X	
		Intervention providers	Focus group		X		X	
		Parents/guardians	Questionnaire and semi-structured interview				X	
		School staff	Semi-structured interview				X	
6. To understand context, i.e. the external factors that affected intervention implementation	**Context**^**a**^ *Political, economic, or social influences on implementation of the innovation*	Intervention recipients	Feedback questionnaire and focus group		X		X	
		Intervention providers	Feedback questionnaire				X	
		Intervention providers	Focus group		X		X	
		Parents/guardians	Questionnaires and semi-structured interviews				X	
		School staff	Semi-structured interviews				X	

Abbreviations: *PA* physical activity, *MVPA* moderate to vigorous intensity physical activity

^a^Based on measure and terminology reported in Pearson et al. [43]

^b^Based on measure and terminology reported in Steckler and Linnan [48]

^c^Applicable to newly enrolled intervention recipients

#### The COVID-19 pandemic implications for this study

In January 2021, there was a nationwide lockdown that involved national school closures. Consequently, although the intervention was originally designed to be delivered in person on school grounds, it was divided into two phases. Both phases were delivered at the same time (4 pm) each week on the same day (Tuesday). Four classes were delivered via Zoom during phase 1 using the online school platform. When schools fully reopened in April 2021, four classes were delivered in person (phase 2) in the school sports hall or playing field. In response to COVID-19-related social restrictions, just 1 year group was invited to participate as ‘intervention recipients’. This was a protective measure to avoid integrating students from different year groups and to accommodate for social distancing (2 m). Furthermore, school guidelines permitted just one researcher (SMQ) to attend the school to collect data during intervention implementation.

### Recruitment and participants

#### Intervention providers (who delivered it)

Intervention providers were six volunteer transition year students (aged 15–17) known as project leaders (*n* = 6). In Ireland, transition year is a 1-year school programme that can be taken in the year after the junior cycle (students aged 12–15) and before the 2-year leaving certificate programme (students aged 15–18) [49]. It is not a standard academic year. Instead, the year is designed around giving students life skills and a more hands-on aspect to learning. The six project leaders who expressed an interest were familiar with the GAP programme from the codesign process [24]. As a recognition for their time and effort, project leaders could use the GAP to contribute towards ‘Gaisce’, a national self-development programme for young people in Ireland between the age of 15 and 25 [50]. At the end of the study, each project leader also received a ‘Girls Active Project’ certificate as an expression of appreciation (Supplementary file 4).

#### Intervention recipients (to whom it was delivered)

Given that national evidence suggests the largest reduction in PA levels for females occur between second (aged 13–14) and third year (aged 14–15) in postprimary school [9], the school principal, physical education teacher and authors agreed to target the second-year students (*n* = 78, aged 13–14). Intervention recipients were recruited over two rounds, with the second round required due to low recruitment experienced at phase online (during COVID-19 school closure). The number of intervention recipients who were invited, consented and completed the questionnaires was recorded at week 1 (phase one baseline), week 8 (phase 2 baseline) and week 12 (post-intervention). The number of recipients who withdrew or were lost to follow-up were recorded at mid-intervention (week 8) and post-intervention (week 12). A standard threshold for study attrition (> 20%) was employed [43, 51].

The GAP intervention programme was pitched to second-year students and their parents/guardians as a fun, free, peer-led exercise programme suitable for all fitness levels that included a variety of activities and provided opportunities to win prizes. Recruitment strategies for both phases included an information email sent from the school to each second-year student and school social media posts on behalf of the research team. ‘Girls Active Project’ posters made by students [24] were displayed in the school prior to school closure (pre-COVID-19 lockdown, December 2020). In addition, a ‘word-of-mouth’ campaign [52] was employed in phase 2 (in person, April 2021). This involved project leaders visiting each of the second-year classrooms with the researcher (SMQ) during school hours to encourage them to participate in the intervention. Project leaders explained the purpose of the GAP intervention programme and answered any questions their peers had.

Adolescent females were eligible to take part in this study if they were (**a**) a project leader or (b) a second-year student. Eligible students received a letter of invitation from the school on behalf of the research team containing information sheets, assent and parental/guardian consent forms and a PA readiness questionnaire for them and their parent/guardian to read and sign. If the parent/guardian answered ‘yes’ to any of the questions in the PA readiness questionnaire, they were advised to talk to their general practitioner to discuss if their daughter is able to participate in the physical requirements of the PA programme. Students were excluded from the study if they had not provided assent or parental/guardian consent or they had been advised by their general practitioner not to undertake PA. Participating students were assigned identification codes to protect identity.

#### Other key stakeholders

At post-intervention, the parent/guardian of each second-year student (*n* = 78) was sent a text message from the school that contained a link to an anonymous online Qualtrics questionnaire [53]. Informed consent was provided online. A reminder text message was sent a week later. Parents/guardians provided their contact details if they were willing to participate in a short, semi-structured, audio-recorded phone call interview. School staff members (two physical education teachers and school principal) were invited to participate in a semi-structured, audio-recorded interview on school grounds at post-intervention. The three school staff members provided written informed consent prior to the interview.

#### Intervention delivery

As a team, intervention providers (project leaders) delivered a 45-min exercise class after school once per week to intervention recipients. Project leaders mutually decided what was delivered (intervention content) and changed it each week. This current study comprised eight classes over a 12-week period (March to May 2021) to accommodate school holidays (2 weeks during April). Training for the project leaders consisted of meetings during school hours, either online or in person, with the physical education teacher and researcher (SMQ) to collaboratively discuss and for project leaders to ultimately decide: what was delivered (for example boxing) and how (for example 5-min warm-up including skipping, 30 min of boxing with intervals of bodyweight exercises, 5 min cool down and stretches). The activities (listed in Table 2 ) were a result of these discussions, and outlines when the 21 behaviour change techniques were employed during this intervention trial. At the start of each phase (week 1 and week 8), the recipients were encouraged to set a goal to attend each week and were informed of the potential rewards for participation, i.e. a signed ‘Girls Active Project’ certificate and entries into a raffle to win prizes, including €20 vouchers. The ‘class plan’ template and certificates can be found in Supplementary file 4. Procedures to standardise delivery were used [54], and a general class structure was followed:

- Welcome and introductions made to intervention recipients and mention the purpose of the GAP programme
- Intervention recipients given a chance to contribute and ask questions
- Exercises explained and demonstrated and intervention recipients given a chance to practise the exercises
- Intervention recipients congratulated for participating and reminded about next week’s class.

**Table 2:** The behaviour change techniques employed during the Girls Active Project intervention trial [23]

Behaviour change technique	Week 1_class 1: HIIT	Week 2_class 2: dance	Week 3_class 3: boxing	Week 4_class 4: dance	Week 8_class 5: boxing	Week 9_class 6: Football	Week 10_Class 7: basketball	Week 11_class 8: dance
Goal setting (behaviour)	X				X			
Action planning	X	X	X	X	X	X	X	
Monitoring of behaviour by others without feedback	X	X	X	X	X	X	X	X
Social support (practical)	X	X	X	X	X	X	X	X
Social support (emotional)	X	X	X	X	X	X	X	X
Instruction on how to perform a behaviour	X	X	X	X	X	X	X	X
Information about health consequences	X	X	X	X	X	X	X	X
Monitoring of emotional consequences	X	X	X	X	X	X	X	X
Demonstration of the behaviour	X	X	X	X	X	X	X	X
Prompts/cues	X	X	X	X	X	X	X	X
Behavioural practice/rehearsal	X	X	X	X	X	X	X	X
Habit formation	X	X	X	X	X	X	X	X
Generalisation of a target behaviour	X	X	X	X	X	X	X	X
Credible source	X				X			
Material incentive (behaviour)	X				X			
Material reward (behaviour)				X				X
Non-specific reward								X
Social reward	X	X	X	X	X	X	X	X
Non-specific incentive	X				X			
Restructuring the social environment	X	X	X	X	X	X	X	X
Verbal persuasion about capability	X	X	X	X	X	X	X	X

It was not mandatory for students to have their cameras on during online classes (phase 1). If project leaders preferred not to deliver the class with their cameras on, a suitable YouTube exercise video was chosen instead. A physical education teacher was present to offer any modifications to exercises if required. Researcher (SMQ) attended each class to assist in supervision and if needed offer encouragement and consultation to project leaders.

### Data collection

Quantitative and qualitative data were gathered to assess intervention feasibility. All data collection tools used can be found in Supplementary file 3.

#### Questionnaires

Recipients were asked to complete a self-reported questionnaire at every data collection time-point (week 1, week 8 and week 12). The questionnaire was piloted and modified with the students who codesigned the intervention [24] to ensure there was no ambiguity in the questions and to identify any potential problems the recipients might experience. At baseline, recipients were asked to complete the questionnaire at home online (week 1, phase 1), via a link to a Qualtrics [53] questionnaire or on paper (week 8, phase 2) administered by the researcher (SMQ) during agreed scheduled school hours. This included a short demographic section capturing date of birth, nationality, disability status and the name of the street they lived on. Street names were mapped against a publicly available ‘deprivation indices’ [55] for Ireland, which identifies underprivileged areas by estimating deprivation on an 8-point scale (1 = extremely affluent to 8 = extremely disadvantaged) [55]. Self-reported outcome measures were captured at every data collection time-point: week 1 (phase one baseline), week 8 (mid-intervention and phase 2 baseline), and week 12 (post-intervention). Previously validated scales were used to measure the outcomes, including minutes of daily moderate-to-vigorous intensity PA (MVPA) [56–58], self-rated health [58], life satisfaction [59], self-efficacy related to PA [60–62] and PA enjoyment [61, 63–65]. Clear instructions were provided in written form to each recipient on how to accurately measure and take note of their height (to the nearest cm) and weight (to the nearest 0.1 kg) at home. Data completion rates are expressed as overall percentages. The mean scores, standard deviations and ranges for each measure are also presented to provide context.

Self-reported feedback questionnaires were developed by the authors to capture stakeholder experiences with various aspects of the intervention. This feedback questionnaire was integrated into the recipients questionnaire at week 8 (mid-intervention) and week 12 (post-intervention). A 5-point Likert scale (1: dislike very much to 5: like very much) was used to assess recipients’ satisfaction with the programme. A predetermined mean score of ≥ 3.5 out of 5 was considered feasible [66]. Recipients were asked to rate factors influencing participation, such as ‘my friends’, using a 5-point Likert scale (1: not at all to 5: extremely). Perceived sustainability was assessed by asking recipients if they would like the programme to remain in the school and if they would continue to participate if it did. Recipients were also asked to share opinions on how to improve the programme. Project leaders were asked to complete a similar feedback questionnaire at post-intervention (week 12).

A total of 20 parents/guardians participated in the online questionnaire (*n* = 20, response rate: 26%). It took approximately 6 min to complete. Parents/guardians of second-year students were asked to explain why they thought their daughter did or did not participate, if they would like for the programme to remain as an option for students attending the school and provide suggestions on what the school could do to make it easier for them as a parent/guardian to enable their daughter to participate. Parents/guardians of intervention recipients indicated their daughter’s satisfaction with the programme by rating various statements, (e.g. my daughter enjoyed participating in the GAP) using a 5-point Likert scale (1: disagree a lot to 5: agree a lot).

#### Attendance and delivery logbooks

Student attendance was recorded and monitored by the physical education teacher and researcher (SMQ). Project leaders were asked to complete a short (approximately 3 min), self-reported provider checklist (‘project leader logbook’) online using a Qualtrics [53] questionnaire after each class that they attended. Project leaders received weekly reminder emails to complete the logbook. The delivery logbook contained an implementation checklist that was developed by the authors. The checklist assessed if the class aims (based on the behaviour change techniques (Table 2)) were delivered that day. A total of 38 logbooks were requested. Intervention fidelity was measured via the degree to which the treatment (exercise classes) was delivered as intended by project leaders. Levels of fidelity previously reported in the literature were applied, with 80–100% interpreted as ‘high’ fidelity, 51–79% as ‘moderate’ and 0–50% as ‘low’ fidelity [67–70]. This study aimed for a benchmark of 80% fidelity. Total fidelity is expressed as an average of the class aims being delivered across the eight exercise classes.

#### Semi-structured focus groups and interviews

Four semi-structured focus groups occurred during school hours, two with intervention recipients and two with project leaders (week 8 and week 12) lasting approximately 20 min (range: 17–26 min). Two parents/guardians participated in a semi-structured phone call interview, lasting on average 9 min (range: 7–11 min). These interviews were intentionally designed and specifically promoted as being short with the intention of increasing parental/guardian engagement. Despite being short, the questions asked complemented the parent/guardian questionnaire and addressed study objectives. Semi-structured interviews with the three school staff members occurred on school grounds at post-intervention lasting on average 26 min (range: 20–33 min).

Topic guides used for the focus groups and interviews were developed by the authors and aimed to explore stakeholders’ satisfaction with the GAP programme and its perceived sustainability in the school and to identify any contextual factors that may have affected implementation. Stakeholders revealed what they liked and disliked about the programme, reasons for participation, and provided recommendations for future implementation. Each semi-structured focus group and interview was audio recorded, and hand-written notes were taken by the researcher (SMQ). The audio recordings were transcribed verbatim, and pseudonyms were assigned to protect participant identity.

### Data analysis

Descriptive analyses were performed using the Statistical Package for Social Sciences (SPSS) version 25. Each proposed outcome measure is represented by a mean score, as well as a rate of completion. Focus group and interview transcripts were analysed using a six-step thematic approach [71]. Initially, each transcript was read and re-read several times by the researcher (SMQ), who developed a sample coding frame. These coded transcripts were reviewed by the remaining authors (SJB, AS and MRS). The coding frame was refined iteratively by SMQ, SJB, AS and MRS with subsequent discussions. Anonymised illustrative quotes supporting emerging themes were highlighted and agreed by researchers.

## Results

### Enrolment, who participated and why? (Objective 1)

**Fig. 1:**
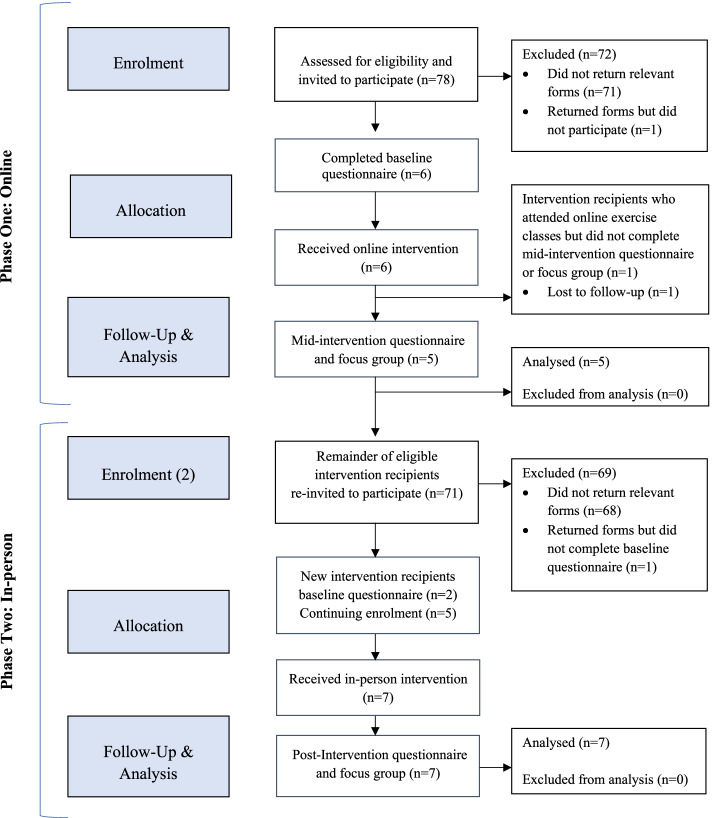
Flow chart of intervention recipients through this study based on the CONSORT 2010 flow diagram [46]

A total of ten second-year students returned the relevant documents to take part. Of which, eight completed the baseline questionnaire (six at week 1 and two at week 8) and participated in the intervention (*n* = 8/78, 10.3%). One student who consented did attend but was absent on both days of data collection. Another student did not respond to emails and was eventually lost to follow-up. Seven recipients completed data collection at week 12, post-intervention (*n* = 7/8, 87.5%) (Fig. 1). During the in person delivered classes (phase 2), the physical education teacher and researcher (SMQ) observed that additional second-year students attended yet failed to return the necessary documents to participate in data collection. Of the eight recipients who did complete the baseline questionnaire, the mean age was 13.25 (range: 13–14, *SD*: 0.46), all of Irish nationality (*n* = 8, 100%) and with no reported disability (*n* = 8, 100%). The deprivation indices based on home street addresses ranged from ‘disadvantaged’ to ‘very disadvantaged’ (mean: 6.4, range: 6 ‘disadvantaged’ to 7 ‘very disadvantaged’, *SD*: 0.52).

Facilitating factors that influenced participation shifted between both phases of the intervention. Improving health and well-being and challenging oneself were the highest rated factors in phase 1 (mean: 4.8, *SD*: .45), whereas being with others was the number one facilitator (mean: 4.8, *SD*: 0.45) during phase 2, followed by wanting to be physically fit (mean: 4.40, *SD*: 0.89). Fun, enjoyment and socialising with peers were identified as key motivators, ‘that’s why I did it, I just went, and I actually really liked it because it’s a nice way to spend time with the girls as well’ (Intervention recipient I, post-intervention focus group). Other facilitators to participation included the desire to feel like they have achieved something and the lack of alternative options during the COVID-19 pandemic. Most parents/guardians believed their daughter participated to improve health and fitness, ‘she wanted to get more active and to be more involved in after school activities’ (parent K, post-intervention questionnaire) and to spend time with peers, ‘I think she participated because she enjoys trying out new things and hanging out with her friends’ (parent J, post-intervention questionnaire). Of less concern, recipients rated the ‘Girls Active Project’ certificate (mean: 1.80, SD: 1.30, phase 1) and family (mean: 2.0, SD: 1.23, phase 2) as the lowest motivating factors. Recipients scored the rewards, including vouchers, as a ‘slightly’ motivating factor in both phases of the intervention (mean: 2.8, *SD*: 0.45, phase 1 vs mean: 2.4, *SD*: .89, phase 2). Despite the project leaders deeming the rewards a success, ‘I think they were amazing. I think they enjoyed it and they definitely appreciated it. They were like ‘I got a voucher for doing something I actually enjoyed’, and then they were like I’m going to keep doing that’ (project leader B, post-intervention focus group), recipients described the rewards as just a ‘bonus’, and not a leading factor influencing involvement.

### Attendance, and was the intervention implemented as intended? (Objective 2)

Project leaders delivered eight exercise classes, and all planned behaviour change techniques were employed. No adverse events were reported. On average, providers attended 79% of classes (71% phase 1 vs 87.5% phase 2). Attendance was slightly lower for recipients (68%), with no significant difference found between phase 1 (67.8%) and phase 2 (68.7%). A total of 31 delivery logbooks (15 at phase 1 and 16 at phase 2) out of a possible 38 were completed (82% completion rate). On average, most of the exercise classes started on time (94%) and were delivered as planned (87%), ‘We gave the class the exact plans as we did in our head, it was great, everyone cooperated brilliantly!’ (project leader F, written extract from logbook phase 2)*.* Project leader absence or an unrelated injury was cited as reasons why a class was not delivered as planned. The implementation checklist results are presented in Table 3. On average, 87% of the aims were delivered (89% phase 1 vs 86% phase 2). During phase 1, project leaders believed the recipients were practising the exercises (aim 5), although said it was difficult to judge because some recipients had their cameras off, ‘I think they were actually doing it, but they were just embarrassed’ (project leader A, mid-intervention focus group).

**Table 3:** Implementation checklist responses^a^ based on project leaders logbooks

Class aim	Phase 1: online (15 logbooks)	Phase 2: in person (16 logbooks)	Total intervention (31 logbooks)
**1. Welcome and introductions were made**	100% yes	100% yes	100% yes
**2. Purpose of the Girls Active Project was mentioned**	93% yes	69% yes	80% yes
**3. Recipients were given a chance to contribute to the discussion and ask questions**	93% yes	100% yes	97% yes
**4. Exercises were explained and demonstrated**	93% yes	88% yes	90% yes
**5. Recipients were given a chance to practice the exercises**	50% yes	75% yes	63% yes
**6. Recipients were congratulated for joining the class and encouraged to be active**	100% yes	94% yes	97% yes
**7. Recipients were reminded about next week’s class**	93% yes	75% yes	83% yes
**Total fidelity**	**89%**	**86%**	**87%**

^a^Options, yes/no/unsure

### Are the proposed outcome measures feasible? (Objective 3)

On average, the rates of completion for the self-reported outcomes were high at 90%. Table 4 provides a summary of the outcome data and completion rates at the three data collection time-points. Weight (kg) and height (m) data were completed the least by recipients at 50% and 80%, respectively. Completion of MVPA levels, self-rated health, life satisfaction and PA self-efficacy were 100% at all data collection time-points.

**Table 4:** Measure completion and scores for proposed self-reported outcome data

Measure	Week 1: baseline (phase 1), online			Week 8: mid-intervention and baseline (phase 2), paper-based			Week 12: post-intervention, paper based			Overall
	Mean (SD)	Range	Percent completed	Mean (SD)	Range	Percent completed	Mean (SD)	Range	Percent completed	Percent completed
**MVPA (7 days)**	3.75 (1.9)	0–5 days	100 (6/6)	4.1 (1.7)	1–7 days	100 (7/7)	4.0 (1.6)	1–7 days	100 (7/7)	**100 (20/20)**
**Height (m)**	1.61 (.07)	1.49–1.68	100 (6/6)	1.65 (.04)	1.60–1.70	57 (4/7)	1.65 (.03)	1.60–1.70	86 (6/7)	**80 (16/20)**
**Weight (kg)**	51.1 (8.5)	44.4–66.0	100 (6/6)	55.6 (14.7)	45.2–66.0	29 (2/7)	53.0 (2.8)	51.0–55.0	29 (2/7)	**50 (10/20)**
**Self-rated health**	3.5 (0.55)	3–4	100 (6/6)	3.3 (0.76)	2–4	100 (7/7)	3.3 (0.76)	2–4	100 (7/7)	**100 (20/20)**
**Life satisfaction**	8.0 (1.3)	7–10	100 (6/6)	7.0 (2.3)	3–10	100 (7/7)	7.0 (2.3)	3–10	100 (7/7)	**100 (20/20)**
**PA self-efficacy**	4.1 (0.42)	3.4–4.5	100 (6/6)	4.0 (0.70)	2.9–4.5	100 (7/7)	4.1 (0.78)	2.9–4.9	100 (7/7)	**100 (20/20)**
**PA enjoyment**	68.2 (13.3)	45–79	100 (6/6)	73.5 (7.8)	60–80	86 (6/7)	74.6 (4.5)	69–80	100 (7/7)	**95 (19/20)**

Scale scores, MVPA (0–7 days); height (metre), weight (kg), self-rated health (1: ‘poor’ to 4: ‘excellent’), life satisfaction (0: ‘worst possible life’ to 10: ‘best possible life’), PA self-efficacy (average of 8 items on a 5-point Likert scale, 1: disagree a lot to 5: agree a lot), PA enjoyment (total scores range from 16: lowest to 80: maximum enjoyment)

Abbreviations: *SD* standard deviation, *MVPA* moderate to vigorous physical activity, *M* metre, *kg* kilogram, *PA* physical activity

### Were the stakeholders satisfied with the intervention? (Objective 4)

Satisfaction levels reported by intervention recipients and project leaders at mid-intervention (week 8) and post-intervention (week 12) for each aspect of the programme are presented in Table 5. Overall satisfaction rates for both recipients and project leaders indicated high acceptability of the intervention. Two aspects of the intervention achieved a mean score of < 3.5 out of 5 and thus considered not feasible. This included the intervention ‘being online’ (mean: 3.4, *SD*: 0.55) and project leaders ‘completing the weekly logbooks’ (mean: 3.3, *SD*: 1.0).

**Table 5:** Satisfaction levels reported by intervention recipients and intervention providers on aspects of the Girls Active Project at mid-intervention and post-intervention

Aspect of the Girls Active Project intervention	Intervention recipients (*n* = 5)		Intervention recipients (*n* = 7)		Intervention providers (*n* = 6)		Categorisation^a^
	Week 8: mid-intervention (phase 1)		Week 12: post-intervention (phase 2)		Week 12: post-intervention (overall)		
	Mean (SD)	Range	Mean (SD)	Range	Mean (SD)	Range	
**Organisation**	4.6 (0.55)	4–5	5.0 (.00)	5–5	4.3 (0.82)	3–5	Feasible
**Class duration**	4.6 (0.55)	4–5	5.0 (.00)	5–5	4.2 (0.41)	4–5	Feasible
**Dates of delivery**	4.8 (0.45)	4–5	4.7 (0.76)	3–5	4.0 (1.1)	2–5	Feasible
**Start and end time**	4.8 (0.45)	4–5	5.0 (.00)	5–5	3.5 (0.55)	3–4	Feasible
**Variety of activities**	4.4 (0.89)	3–5	4.9 (0.38)	4–5	4.8 (0.41)	4–5	Feasible
**Delivery of activities (project leaders)**	4.8 (0.45)	4–5	4.9 (0.38)	4–5	NA	NA	Feasible
**Information provided**	4.6 (0.55)	4–5	4.6 (0.79)	3–5	NA	NA	Feasible
**It being online**	3.4 (0.55)	3–4	NA	NA	NA	NA	Not feasible
**It being in person**	NA	NA	5.0 (.00)	5–5	NA	NA	Feasible
**Being a leader delivering the classes**	NA	NA	NA	NA	4.8 (0.41)	4–5	Feasible
**Completing the weekly logbooks**	NA	NA	NA	NA	3.3 (1.0)	2–5	Not feasible
**Working as part of a team**	NA	NA	NA	NA	4.7 (0.52)	4–5	Feasible
**Overall satisfaction**	5.0 (.00)	5–5	4.7 (0.76)	3–5	4.7 (0.52)	4–5	Feasible

Scale scores: 5-point Likert scale (1: dislike very much to 5: like very much)

Abbreviations: *SD* standard deviation, *NA* not applicable

^a^A predetermined mean score of ≥ 3.5 out of 5 was considered feasible

Seven parents/guardians of second-year students answered ‘yes’ to their daughter taking part in the intervention (*n* = 7/15, 47%). Most agreed that their daughter enjoyed the programme (mean: 4.7, *SD*: 0.82, range: 3–5); liked that it was peer led (mean: 4.4, *SD*: 0.89, range: 3–5) and offered variety (mean: 4.6, *SD*: 0. 55, range: 4–5); and liked the timing, dates and duration of the classes (mean: 4.8, *SD*: 0. 50, range: 4–5).

Overall, the qualitative data also suggested that stakeholders were satisfied with the intervention. Both recipients and project leaders preferred the intervention delivered in person, ‘it went a lot better than I thought it would online, but I preferred it face-to-face’ (project leader B, post-intervention focus group). Project leaders did not indicate any dissatisfaction with completing the logbooks, ‘Just the survey? Oh, that was fine, got that done in like 5 minutes’ (project leader D, post-intervention focus group). For one project leader, the timing and dates coincided with other commitments; however, they recognised that ‘it’s really hard to find a time that suits everyone’ (project leader E, post-intervention focus group). Project leaders enjoyed working with peers and learning new skills ‘we learned how to take initiative, like if stuff goes wrong, we adapted and planned different classes and stuff’ (project leader A, post-intervention focus group) and their leadership role, ‘it gave us a leadership role in the school for sure. It made you feel included, and it made you feel like you had a place to go after school where everyone had the same interests, and you were all there for the same reason’ (project leader D, post-intervention focus group). To improve intervention acceptability, project leaders recommended to extend class duration and provide extra time at the start of the class to setting up, ‘we could try set up everything before second years [recipients] arrive so that we are prepared, and it doesn’t take up class time’ (project leader E, feedback questionnaire). Additional promotional activities from the school to encourage participation was also suggested, ‘the school didn’t really like promote it that much, like they could have posted it more and put it on the Instagram pages, I think that would have of like helped more people come’ (project leader A, post-intervention focus group).

Recipients disliked that not many students enrolled and equally suggested that the school further promotes the GAP programme through weekly reminders, more posters and announcements via the school intercom and social media posts, ‘put up a few pictures or a link or if there was a little video put together of what one class is literally like’ (intervention recipient D, post-intervention focus group). Recipients described how they enjoyed the exercise classes because they got to spend time with friends, relieve stress, did not feel judged and liked that the project leaders were close in age. They also expressed their satisfaction with the variety of activities offered as it introduced them to new activities, ‘they’re things that like I wouldn’t pick myself but then when I done it, it was good’ (intervention recipient B, mid-intervention focus group) and kept it fun, ‘if it was the same every week, it would just get boring like’ (intervention recipient C, post-intervention focus group). In the phone call interview, one parent/guardian described how it was the variety that attracted her daughter to participate, ‘it was because you were doing a bit of something different every week. That’s what made her, that’s what appealed to her’ (parent 2, interview). Both parents/guardians suggested additional encouragement from the school would increase participation and improve its acceptability, ‘I suppose if they could point out that it was more fun than anything, you know? It wasn’t like it was work’ (parent 2, interview).

School staff members also liked that the programme provided students with an opportunity to try new activities, ‘I think when you give those opportunities then of doing a variety of things where they get the opportunity to try them out, it’s huge because then that could be their thing that they actually go on to do’ (school staff member A, interview). They believed the students involved enjoyed it, ‘you can see them laughing and joking, and that’s what I liked about it, it’s simple and effective and they come away from it happy’ (school staff member B, interview) and particularly liked that, unlike many other extra-curricular PA programmes, the emphasis was not on competition or performance, ‘it was about getting involved and just enjoying physical activity, which I really enjoyed that aspect of it’ (school staff member C, interview). Staff members expressed satisfaction with the intervention being peer-led ‘it’s great because I think we have students that have a great ability to lead and they obviously look up to each other, especially the younger years to the older years’ (school staff member A, interview) and cited many positive impacts the intervention had on the students involved, alongside being active, such as socialising, gaining a sense of belonging, and particularly for project leaders, to feel trusted and develop leadership skills.

To enhance intervention acceptability, staff members recommended extending class duration from 45 min to 1 h, creating more reminders and introducing different rewards to maintain student involvement, such as a ‘Girls Active Project’ t-shirt. Staff members considered the intervention acceptable yet were open to modifications, ‘I think as it goes on in the future, we’ll see where it can be developed as well when it’s more established in the school. And then if there are tweaks and things that need changing, we could do it then’ (school staff member C, interview).

### Was the intervention perceived as compatible with the school setting? (Objective 5)

All project leaders (100%, *n* = 6/6), most parents/guardians (92%, *n* = 12/13) and most recipients (80%, *n* = 4/5 phase 1 vs 85.7%, *n* = 6/7 phase 2) wanted the programme to remain in the school. Project leaders perceived the programme as an appropriate fit, ‘I loved it and it would be a great thing to keep in the school’ (project leader C, feedback questionnaire), with six of them (100%, *n* = 6/6) reporting that they would recommend becoming a project leader to other students ‘I would definitely recommend it as it is an amazing, fun and rewarding experience and not only does it develop your fitness skills, but your leadership ones also’ (project leader E, feedback questionnaire). More recipients reported that they would continue to participate if it were delivered in person than online (85.7%, *n* = 6/7 phase 2 vs 60%, *n* = 3/5 phase 1). During the post-intervention focus group, some of the recipients explained how they never participated in other after-school PA programmes because they perceived them to be competitive in nature. If given the chance, however, they would continue to participate in the GAP programme because it was significantly less competitive, ‘this was a lot more chill’ (intervention recipient C, post-intervention focus group). Likewise, staff members acknowledged how the programme attracted students that were not usually involved in extra-curricular PA clubs, ‘it’s the same girls all the time doing the same sports, whereas the GAP actually opens up exercise to a bigger cohort because you’re not attracting the same people, which is good’ (school staff member B, interview). They perceived this as important as it offered an opportunity for the ‘noncompetitive’ students to engage with PA, ‘the non-competitive people, they need to have something. I mean, those are the people you’re trying to keep involved and to get active, so yeah, absolutely, I think it [the GAP programme] needs to be as fundamental as the other sports, 100%’ (school staff member A, interview). School staff members were optimistic about the intervention’s long-term potential in the school. One staff member believed it was less administratively demanding and required limited involvement in terms of class delivery when compared to other after-school PA programmes, ‘it’s easier. I think it gets better results for less time. So, why wouldn’t you do that?’ (school staff member B, interview). The perceived sustainability of the intervention was also influenced by student enjoyment, ‘I think it’s also far more likely that they continue doing physical activity if it’s enjoyable in the first place. So, as a lifelong learning thing, I think it’s far more sustainable, even than playing on the school team, because lots of people do that for a year or two and then they drop off anyway even if they were sporty. So, from the point of view of enjoyment and doing it from a health promoting point of view, I think this was far more sustainable and I think they’re likely to go with it’ (school staff member C, interview).

### What were the external factors that affected intervention implementation? (Objective 6)

#### Barriers

There were external barriers to recruitment. Stakeholders acknowledged that employing recruitment strategies was difficult due to the COVID-19 pandemic, especially during school closure, ‘it’s extremely hard to do in COVID. Besides emailing and telephone calling, and getting them to talk to their friends like, they’re not in school’ (school staff member B, interview). Recruiting and engaging students online were identified as barriers, ‘initially it had to be online you know, and that doesn’t really work for a lot of our students, as we learned’ (school staff member C, interview). Staff members stated that many students did not attend compulsory academic classes online; therefore, anything extra-curricular was considered increasingly challenging. Additionally, project leaders suggested that the intervention being online was a barrier to participation, ‘you’re tired from doing online school and then you just want to take a break after it, and then it’s hard to go back online to do it’ (project leader A, post-intervention focus group). Recipients reported homework and distinct to phase 1 (online), failing to remember ‘quarantine brain got the best of everyone’ (intervention recipient B, mid-intervention focus group) as barriers to participation.

The COVID-19 pandemic continued to affect intervention implementation during phase 2 (in person). Project leaders felt constrained, ‘even now in school we’re limited in what we can do because of Corona’ (project leader B, mid-intervention focus group). The eight parents/guardians of second-year students whose daughter did not take part in the intervention (*n* = 8/15, 53%) listed homework, other commitments such as music, a dislike for after-school activities and anxiety and fears surrounding the COVID-19 pandemic as barriers to enrolment, ‘she is finding it hard to settle back into school since lockdown, with her anxiety of school’ (parent B, questionnaire).

#### Facilitators

In contrast to the above, some recipients also considered the COVID-19 pandemic as an external facilitator to enrolment due to the lack of other PA options available, ‘like this was definitely a good option like you know what I mean, something to do when everything else was closed’ (intervention recipient A, mid-intervention focus group). Project leaders could also recognise COVID-19 as a facilitator to enrolment, ‘loads of the places weren’t open at the time to go and do your exercises’ (project leader D, post-intervention focus group). Staff members identified the school reopening (phase 2, April 2021 post lockdown) as an external factor that positively affected intervention implementation as it allowed for the ‘word of mouth’ recruitment strategy to be implemented, ‘the numbers definitely increased over the weeks and when they’d be in class talking, they’d talk to each other and encourage each other to go, which is… that’s huge’ (school staff member B, interview). Staff members believed that with time, enrolment into the in-person delivered PA programme would have increased. Other identified facilitators included the following: stakeholder buy-in via the schools’ steady support and commitment, including use of the online platforms and sport hall facilities; the positive working relationship and regular communication with the research team; and the project leaders, ‘they’re confident students, they’re enthusiastic. So, you know, that’s a huge strength in a way’ (school staff member A, interview).

## Discussion

This paper presents the reach, dose, fidelity, acceptability and compatibility of the peer-led, after-school GAP intervention programme, the data completion rates of proposed self-reported outcome measures and identifies the external factors to implementation. The peer-led, after-school PA programme was trialled for the first time over a 12-week period with disadvantaged adolescent females in a single-sex, female-only, designated disadvantaged postprimary school in Ireland during the COVID-19 pandemic. This study encountered significant contextual barriers and challenges with recruitment. The in-person delivered programme, however, shows promise as an intervention that can be feasibly implemented and evaluated. Results indicated the intervention was implemented as intended and was deemed acceptable and compatible with the school setting. The following paragraphs discuss the strengths and challenges of this trial and provide recommendations for future research.

### Strengths

Retention rates were high, providing support for the feasibility of this trial. Peers, enjoyment, improving health and fitness and the wide variety of activities offered were regarded as essential for initiating and maintaining interest. The logbooks signified that the exercise classes were delivered with high fidelity (> 80%), suggesting that the intervention was delivered as intended. These results, however, must be interpreted with caution since, as often required [72], the fidelity measures used were developed specific to this intervention, making it difficult to use valid and reliable fidelity measures. The average dose received by recipients (68%) compared positively to similar feasibility studies on after-school PA interventions for adolescent females, such as the Girls-Peer Activity (G-PACT) project [32] which recorded an average of 40% and 47% attendance in each ‘class’ and ‘choice’ school, respectively, and the Bristol Girls Dance Project [73] with an average of 13.3 sessions attended out of a maximum of 18 (74%). This was a positive finding given that previous reviews [16, 20] on after-school PA programmes have emphasised the importance of high programme attendance rates as the effectiveness of a programme strongly depended on attendance. In general, completion rates for the proposed self-reported outcome measures were high. This demonstrated recipients’ comprehension of the proposed measures and could indicate the feasibility of using these outcome measures in a future study.

Positive stakeholder responses were particularly welcome, given the unprecedented circumstances of the COVID-19 pandemic during intervention implementation. Consistent with previous school-based PA interventions for adolescent females [74, 75], the stakeholders in this study acknowledged the programme’s potential to engage inactive students and provide an alternative option to students who may not be attracted to the competitive and performance-focused nature of traditional extra-curricular PA programmes. The stakeholders also recognised the positive impact the programme had on the students involved, alongside being active, such as skill development, which is likely to have implications for ongoing delivery. The benefits associated with being a student peer ‘mentor’ or ‘leader’ have been documented in past peer-led PA interventions [74, 76, 77].

Despite recent findings to support the use of online interventions to encourage adolescents engagement with PA [78], given that the GAP programme was designed and originally intended to be delivered in person after school in the school setting, it was perhaps unsurprising that the in-person delivered programme (phase 2) was deemed more acceptable than online (phase 1). Since time and staff availability are recognised barriers to the implementation of PA policies in schools [30], it was promising to discover that this peer-led intervention was considered easier to manage and less administratively demanding than other extra-curricular PA programmes. High intervention compatibility was also likely to be due to the codesign work [24] previously conducted with students in the school. Importantly, this process enabled the programme to be embedded within the school curriculum and allowed for project leaders’ time to contribute towards the already established ‘Gaisce’ award [50]. Steady support and commitment from the school, and the enthusiastic project leaders, were cited as factors that helped intervention implementation. This highlighted the importance of stakeholder buy-in, as found in previous school-based PA initiatives [79, 80]. Finally, tailoring interventions to the individual school context is important for scaling up [79, 81]. Stakeholders’ willingness to modify the intervention suggested that they were engaged and felt empowered to tailor the programme to suit their context. This in itself is a positive finding.

### Challenges

Despite two rounds of recruitment, enrolment was low. Recruitment is crucial to the success of research programmes as high enrolment rates demonstrate that the programme reached the population for which it was designed. Adolescent females, particularly from disadvantaged groups [82, 83], however, can be a difficult population to reach. The challenges of recruiting adolescent females into PA interventions have been recognised in previous research [32, 52, 84]. 

This study encountered additional contextual barriers to recruitment attributable to the COVID-19 pandemic. Employing recruitment strategies during school closure (phase 1) was difficult given students were not present in school and the lack of engagement with ‘online school’. Another factor which may have proven to impact recruitment is that after-school PA programmes are often available to all students in the school or split into junior (first, second and third-year students aged 12–15) and senior cycle (4th, 5th and 6th-year students aged 15–18). As previously discussed, just 1 year group was invited to take part in this study. This would be unusual school practice and could have made the recruitment process more difficult. It was observed that additional students attended the in-person programme (phase 2) yet failed to return the relevant documents to participate in data collection. The perceived burden of returning documents could be a reason for this. Student absenteeism was a barrier to data collection identified in this study. A future trial could encounter similar challenges given that evidence suggests lower socio-economic status is associated with higher levels of absenteeism at school among adolescent females [85]. Despite the fact that the rewards used in this study (certificates and vouchers) were selected by students in the codesign process [24], recipients did not in actuality perceive these rewards as strong motivators influencing participation.

The two proposed self-reported outcome measures least completed by recipients were weight and height data. This could indicate that despite the detailed written instructions provided, requesting recipients to record these measurements at home and complete the questionnaires at school was not feasible. Project leaders deemed completing weekly logbooks as unfeasible in the feedback questionnaires; however, they did not indicate any dissatisfaction with the task in the focus groups. The online programme (phase 1) was also deemed unacceptable by intervention recipients. Most stakeholders agreed that phase 1 (online) went better than anticipated but found phase 2 (in person on school grounds) more acceptable and sustainable. This unique aspect to the study was necessary due to school closure resulting from the COVID-19 pandemic. Ultimately, similar to O’Kane et al. [86], who reported challenges with remote data collection during COVID-19 lockdown, phase 1 of this current study (online) was largely dependent on students’ engagement with home learning.

### Recommended refinements

Low enrolment rates in this study could suggest current recruitment strategies were ineffective; thus, further work is required to develop and test recruitment methods. This could involve codesigning strategies with students, parents and school staff to ensure the revised strategies are relevant, acceptable and practical. The ‘word of mouth’ campaign used in phase 2 was considered valuable by stakeholders in this study; however, students recommended to further boost programme awareness via school announcements and additional posts on social media, including short videos. Similar to Jago et al. [52], stakeholders in this study recommended extra emphasis on enjoyment of classes when pitching the programme to increase participation. Using a parental/guardian, passive (opt-out) approach has allowed for greater recruitment of adolescents in previous low-risk, nonintrusive, school-based PA interventions [32, 34, 44, 74, 79], such as the Girls-Peer Activity (G-PACT) [32] (94% using passive consent vs 26% using active consent) and the GoActive programme [87] (78% using passive consent vs 23% using active consent). This refinement, however, is dependent on ethical approval from the institutional research ethics board and agreement from the participating school. Another viable approach to recruitment [52] includes providing females the opportunity to sample an exercise class in a ‘taster session’ before committing to the programme. This could enable students to understand what the GAP programme would involve, without the pressure of signing up.

The motivating factors to participation identified in this study, such as being with others and a desire to improve health and fitness, could also prove useful when revising recruitment strategies for a future trial. Indeed, given that friend involvement can be an important factor affecting PA participation [25, 26], recruiting friend groups could be used as a potential strategy [84, 88] to increase recruitment. Other school-based PA interventions have found merit in providing small rewards to facilitate sustained involvement [73, 76], such as sports bags or pens [76]. One stakeholder in this study suggested using different rewards, such as a ‘Girls Active Project’ t-shirt. Further work with adolescent females to identify what rewards, if any, are acceptable and desirable is warranted to potentially improve recruitment and retention in a future trial.

As per usual school practice for after-school PA programmes, a future trial, if permitted, should invite additional year groups to take part. Proposed strategies to improve research with socially disadvantaged groups, such as using flexible data collection methods [82], may need to be considered as a means to increase level of participation in data collection. The PA questionnaire used in this study to assess the attainment of PA guidelines had a high completion rate (100%) across all time points. This approach should be combined with an objective measure, such as accelerometers, to examine the intervention’s potential impact on increasing MVPA levels [89, 90].

Given the low completion rate for self-reported weight, a future trial should reconsider using weight and height measures. Body image has been previously cited by adolescent females, especially those with low perceived competence and high weight status, as an internal barrier to PA participation [91]. In contrast, Demetriou et al. [20] found that females were more receptive than males to after-school PA interventions that promoted weight control. Further discussions with stakeholders could be beneficial in deciding if to include weight and height (either self-reported and/or objective) measures in a future trial. Additionally, further work with project leaders is necessary to revise the delivery logbooks to improve acceptability, while future research could assess the feasibility of using direct observation to monitor fidelity. Evidence suggests observational measures [54, 92, 93] or using a mixed-methods approach (audio recordings, direct observation and self-reported checklists) [70] to assess fidelity of intervention delivery may provide a more insightful understanding of fidelity and its influencing factors. Furthermore, alongside revising recruitment strategies, stakeholders suggested extending class duration from 45 min to 1 h, allocating extra time for project leaders to set-up at school prior to the exercise class and creating more reminders for recipients to attend class.

### Strengths and limitations of this study

There are strengths to this study. This paper contributes to the expanding literature on feasibility studies of school-based PA interventions. Although unintended, the two-phase delivery of the intervention during COVID-19 allowed us to compare feasibility between an online and in-person delivered after-school PA intervention. This feasibility study used a mixed-methods approach and included multiple stakeholders perspectives (i.e. intervention recipients, providers, parents/guardians, physical education teachers and school principal). This allowed us to collect rich meaningful data on feasibility and acceptability of the intervention. Another strength of this study are the learnings and practical recommendations provided for future research.

There are a number of limitations to this study. Although it is acceptable given that this study’s primary aim was to evaluate feasibility, this study involved a small sample size, and therefore, caution in generalisation is warranted. This study did not include an economic evaluation. Assessing the feasibility of using an objective measure (e.g. accelerometers) to capture adolescent MVPA levels was not possible due to school closure and travel restrictions. This study used self-reported PA data, which is dependent on students’ recall ability [89, 90]. Furthermore, while many of the evaluation tools used in this study allowed for specificity, a limitation of this approach is the lack of evidence for the reliability or validity of the scores that such scales generate. This limitation has been acknowledged in similar evaluations of school-based adolescent PA interventions [72, 79].

## Conclusion

The in-person delivered intervention was well-received by stakeholders involved and shows promise as an intervention that can be feasibly implemented and evaluated. Despite the COVID-19 pandemic hindering intervention implementation, classes were delivered as intended, and retention was high. Enrolment, however, was low, amplifying the need for further work on revising and testing recruitment strategies. There were important lessons to be learned from this study, both with and without the lens of the COVID-19 pandemic. This paper contributes to the growing body of knowledge on feasibility studies of after-school PA interventions, where the sharing of this detailed feasibility work may benefit other researchers in reusing techniques that have proved successful or in avoiding similar challenges. The novel GAP intervention programme should be revised using the recommendations from this study, before continuing to a more robust evaluation.

## Supplementary Information

**Additional file 1: Supplementary file 1.** GAP Feasibility Trial_CONSORT checklist

**Additional file 2: Supplementary file 2.** GAP TIDieR checklist

**Additional file 3: Supplementary file 3.** Data Collection Tools

**Additional file 4: Supplementary file 4.** GAP Materials

## Data Availability

The datasets during and/or analysed during the current study are available from the corresponding author on reasonable request.

## Abbreviations

GAP: Girls Active Project
PA: Physical activity
MVPA: Moderate-to-vigorous intensity physical activity
BCW: Behaviour change wheel
BCTs: Behaviour change techniques
