# Supplementary material for: Feasibility of a peer-led, after-school physical activity intervention for disadvantaged adolescent females during the COVID-19 pandemic: results from the Girls Active Project (GAP)
Source: Pilot Feasibility Stud. 2022 Aug 30;8:194. doi: 10.1186/s40814-022-01149-2 (PMC9425823; doi:10.1186/s40814-022-01149-2)
Supplement: Supplementary file 2 — Additional file 2: Supplementary file 2. GAP TIDieR checklist [file 40814_2022_1149_MOESM2_ESM.pdf]

Supplementary File: Girls Active Project Feasibility Study

Table: The Girls Active Project details: in accordance with the Template for Intervention Description and Replication (TIDieR) checklist

Item number	Item	Where it is located
**1**	**Brief name** Provide the name or phrase that describes the intervention.	5
**2**	**Why** Describe the rational, theory, or goal or the elements essential to the intervention.	5
**3**	**What** *Materials:* describe any physical or informational materials used in the intervention, including those provided to participants or used in intervention delivery or in training of intervention providers. Provide information on where the materials can be accessed (online appendix, URL).	10-11
**4**	*Procedures:* describe each of the procedures, activities, and/or processes used in the intervention, including any enabling or support activities.	10-12
**5**	**Who provided** For each category of intervention provider (e.g. psychologist, nursing assistance), describe their expertise, background and any specific training given.	8+10
**6**	**How** Describe the models of delivery (e.g. face-to-face or by some other mechanism, such as internet or telephone) of the intervention and whether it was provided individually or in a group.	7+10
**7**	**Where** Describe the type(s) of location(s) where the intervention occurred, including any necessary infrastructure or relevant features.	6-7
**8**	**When and how much** Describe the number of times the intervention was delivered and over what time period including the number of sessions, their schedule and their duration, intensity or dose.	10-11
**9**	**Tailoring** If the intervention was planned to be personalised, titrated or adapted, then describe what, why, when and how.	n/a
**10**	**Modifications:** If the intervention was modified during the course of the study, describe the changes (what, why, when and how).	7
**11**	**How well** *Planned:* if intervention adherence or fidelity was assessed, describe how and by whom, and if any strategies were used to maintain or improve fidelity, describe them.	14
**12**	**How well** *Actual:* if intervention adherence or fidelity was assessed, describe the extent to which the intervention was delivered as planned.	19-20
