# Supplementary material for: Feasibility of a peer-led, after-school physical activity intervention for disadvantaged adolescent females during the COVID-19 pandemic: results from the Girls Active Project (GAP)
Source: Pilot Feasibility Stud. 2022 Aug 30;8:194. doi: 10.1186/s40814-022-01149-2 (PMC9425823; doi:10.1186/s40814-022-01149-2)
Supplement: Supplementary file 3 — Additional file 3: Supplementary file 3. Data Collection Tools [file 40814_2022_1149_MOESM3_ESM.pdf]

Girls Active Project: Project Leaders Logbook – Online

Girls Active Project

Hi GAP Project Leader!

Thanks again for being a leader and playing a key role in making your school more active.

Please answer the following questions about today's exercise class. It won't take too long.

Remember there are no right or wrong answers and please be as honest as you can.

Q1 Todays Date (dd/mm/yyyy):

____

Q2 Your Name

____

Q3 Did the exercise class start on time? (4pm Tuesday)

- ○ Yes (1)
- ○ No (2)

Q4 Was the exercise class delivered as planned?

- ○ Yes (1)
- ○ No (2)

Display This Question:

If Q4 = Yes

Q5 If yes, give details:

____

____

____

____

____

Display This Question:

If Q4 = No

Q6 If no, why not?

____

____

____

____

Q7 Please indicate whether or not the following factors were addressed or completed during today's class:

	Yes (1)	No (2)	Unsure (3)
Welcome and Introductions were made (1)	o	o	o
Purpose of the Girls Active Project was mentioned (2)	o	o	o
Second years were given a chance to contribute to the discussion and ask questions (3)	o	o	o
Exercises were explained and demonstrated (4)	o	o	o
Second years were given a chance to practice the exercises (5)	o	o	o
Second years were congratulated for joining the class and encouraged to be active (6)	o	o	o
Second years were reminded about next week's class (7)	o	o	o

Q8 Thank you for completing the form.

Any further comments, recommendations or observations you'd like to add, please write them here:

____

____

____

____

____

End of Block: Evaluation

Intervention Recipients Attendance

**ID**	**Record of Attendance (mark with √)**<br>**Exercise Class**									**Notes**
	1	2	3	4	5	6	7	8	Total	
	Date	Date	Date	Date	Date	Date	Date	Date		
A										
B										
C										
D										
E										
F										
G										
H										
I										
J										
**Total**										

Project Leaders Attendance

**ID**	**Record of Attendance (mark with √)**<br>**Exercise Class**								**Total**
	1	2	3	4	5	6	7	8	
A									
B									
C									
D									
E									
F									
**Total**									

Girls Active Project (GAP)

Intervention Recipients: Instructions for Physical Measures

Height Protocol

Record in Centimetres (cm) please

Equipment: Measuring tape

1. Remove shoes
2. If the hairstyle affects your height, adjust it for the test
3. Stand with heels and toes together
4. Arms loosely by your side and back straight
5. Look straight ahead
6. Take a deep breath and stand as straight as possible without your heels lifting off the ground.

Weight Protocol

Record in Kilograms (kg) please (to the nearest .1kg)

Equipment: Scales

1. Wear only light garments
2. Remove items such as your phone and keys from pockets
3. Remove shoes.
4. Stand on the scales, with both feet fully on the weighing platform, heels towards the back edge, and arms loosely by your side.
5. Remain as still as possible with your head facing forward.
6. Step down from the scale.

Please record your height (cm) and weight (to the nearest .1kg). You will be asked to answer this in the questionnaire. Thank you very much for taking part.

Researchers contact details: Sara McQuinn, sara.mcquinn2@mail.dcu.ie, 087-6121709

Intervention Recipients: Baseline Questionnaire

Welcome to the Girls Active Project

Remember:
1. There are no right or wrong answers - this is not a test.
2. Please answer all the questions as honestly and accurately as you can - this is very important.

The information gathered will be kept strictly confidential. No identifying information about you will ever be published or shared.

You can play 2 or 3 of your favourite songs, drink a cup of tea and complete the questionnaire (it will take approximately 10 minutes to complete).

Today's Date: (dd/mm/yyyy)

____

Q1.1 Full Name:
(this will be replaced with an I.D. Code)

____

Q1.2 Date of Birth: (dd/mm/yyyy)

____

Q1.3 Nationality:

____

Q1.4 Do you have a disability?

○ Yes

○ No

○ Rather not say

Q1.5 What is the name of the Street you live on?

Do not give your house number.
For example, XXX etc.

____

You were provided with instructions and asked to measure your height (in centimetres) and weight (in kilograms) at home. Please note your measurements below:

Q2.1 Height (cm): *(leave blank if you'd rather not say)*

____

Q2.2 Weight (kg), to the nearest .1kg: *(leave blank if you'd rather not say)*

____

Read the following statements before answering the two questions below.
Physical activity is any bodily movement.
Physical activity includes: Exercises - Weight training, aerobics, jogging, dancing, etc. Sports - Hurling, football, athletics, swimming, etc. General - Brisk walking, washing the car, walking, or cycling to school, etc. It can be done at different levels of effort:

Moderate Effort makes your heart rate and breathing rate faster than normal. You may also sweat a little. Brisk walking and jogging are good examples.

Vigorous Effort makes your heart rate much faster, and you have to breathe deeper and faster than normal. You will probably sweat. Playing football or tennis are good examples.

Please try to think carefully and be as accurate as possible with your answers.
For these next two questions, add up all the time you spend in physical activity each day. Only include activities of either MODERATE or VIGOROUS effort (as described above).

Q3.1 Over the past 7 days, on how many days were you physically active for a total of at least 60 minutes per day? Please tick one number.

0 days	1 day	2 days	3 days	4 days	5 days	6 days	7 days
◯	◯	◯	◯	◯	◯	◯	◯

Q3.2. Over a typical or usual week, on how many days are you physically active for a total of at least 60 minutes per day? Only include activities of either MODERATE or VIGOROUS effort. Please tick one number.

0 days	1 day	2 days	3 days	4 days	5 days	6 days	7 days
◯	◯	◯	◯	◯	◯	◯	◯

The following questions will ask you about your health and well-being.

Q4.1 Would you say your health is...?

- ◯ Excellent
- ◯ Good
- ◯ Fair
- ◯ Poor

Q4.2 Here is a ladder. The top of the ladder "10" is the best possible life for you and the bottom "0" is the worst possible life for you.
In general, where on the ladder do you feel you stand at the moment?

- ◯ 10 Best possible life
- ◯ 9
- ◯ 8
- ◯ 7
- ◯ 6
- ◯ 5
- ◯ 4
- ◯ 3
- ◯ 2
- ◯ 1
- ◯ 0 Worst possible life

Q5.1 On a scale of 1 (Disagree a lot) to 5 (Agree a lot), please read the below statements and select the answer you feel is most appropriate to you:

	Disagree a lot (1)	Disagree a little (2)	Neither Agree nor Disagree (3)	Agree a little (4)	Agree a lot (5)
I can be physically active during my free time on most days.	○	○	○	○	○
I can ask my parent or other adult to do physically active things with me.	○	○	○	○	○
I can be physically active during my free time on most days even if I could watch TV or play video games instead.	○	○	○	○	○
I can be physically active during my free time on most days even if it is very hot or cold outside.	○	○	○	○	○
I can ask my best friend to be physically active with me during my free time on most days.	○	○	○	○	○
I can be physically active during my free time on most days even if I have to stay at home.	○	○	○	○	○
I have the coordination I need to be physically active during my free time on most days.	○	○	○	○	○
I can still be physically active during my free time on most days no matter how busy my day is.	○	○	○	○	○

Q5.2 When I am active...

	Disagree a lot (1)	Disagree a little	Neither Agree nor Disagree	Agree a little	Agree a lot (5)
... I enjoy it	○	○	○	○	○
... I feel bored.	○	○	○	○	○
... I dislike it.	○	○	○	○	○
... I find it pleasurable	○	○	○	○	○
... it's no fun at all.	○	○	○	○	○
... it gives me energy	○	○	○	○	○
... it makes me depressed	○	○	○	○	○
... it's very pleasant	○	○	○	○	○
...my body feels good	○	○	○	○	○
... I get something out of it	○	○	○	○	○
... it's very exciting	○	○	○	○	○
... it frustrates me.	○	○	○	○	○
... it's not at all interesting.	○	○	○	○	○
... it gives me a strong feeling of success	○	○	○	○	○
... it feels good	○	○	○	○	○
... I feel as though I would rather be doing something else.	○	○	○	○	○

Thank you for taking the time to complete this questionnaire.
Your response has been recorded.

We hope you enjoy the Girls Active Project!

Girls Active Project

Intervention Recipients: Mid-Questionnaire

Remember:

1. There are no right or wrong answers - this is not a test.
2. Please answer all of the questions as honestly and accurately as you can - this is very important.

The information gathered will be kept strictly confidential. No identifying information about you will ever be published or shared.

You can play 2 or 3 of your favourite songs, drink a cup of tea and complete the questionnaire (it will take approximately 10 minutes to complete).

Today’s Date (dd/mm/yyyy):

____

Q1.1 Name:
(this will be replaced with an I.D. Code)

____

You were provided with instructions and asked to measure your height (in centimetres) and weight (in kilograms) at home.
Please note your measurements below:

Q2.1 Height (cm): ________________ *(leave blank if you'd rather not say)*

Q2.2 Weight (kg) to the nearest .1kg: ________________ *(leave blank if you'd rather not say)*

Read the following statements before answering the two questions below.
Physical activity is any bodily movement.

Physical activity includes: Exercises - Weight training, aerobics, jogging, dancing, etc. Sports - Hurling, football, athletics, swimming, etc. General - Brisk walking, washing the car, walking or cycling to school, etc. It can be done at different levels of effort:

Moderate Effort makes your heart rate and breathing rate faster than normal. You may also sweat a little. Brisk walking and jogging are good examples.

Vigorous Effort makes your heart rate much faster, and you have to breathe deeper and faster than normal. You will probably sweat. Playing football or tennis are good examples.

Please try to think carefully and be as accurate as possible with your answers.
For these next two questions, add up all the time you spend in physical activity each day. Only include activities of either MODERATE or VIGOROUS effort (as described above).

Q3.1 Over the past 7 days, on how many days were you physically active for a total of at least 60 minutes per day? Please tick one number.

0 days	1 day	2 days	3 days	4 days	5 days	6 days	7 days
○	○	○	○	○	○	○	○

Q3.2 Over a typical or usual week, on how many days are you physically active for a total of at least 60 minutes per day? Only include activities of either MODERATE or VIGOROUS effort.
Please tick one number.

0 days	1 day	2 days	3 days	4 days	5 days	6 days	7 days
○	○	○	○	○	○	○	○

The following questions will ask you about your health and well-being.

Q4.1 Would you say your health is...?

- ○ Excellent
- ○ Good
- ○ Fair
- ○ Poor

Q4.2 Here is a ladder. The top of the ladder "10" is the best possible life for you and the bottom "0" is the worst possible life for you.
In general, where on the ladder do you feel you stand at the moment?

- ○ 10 Best possible life
- ○ 9
- ○ 8
- ○ 7
- ○ 6
- ○ 5
- ○ 4
- ○ 3
- ○ 2
- ○ 1
- ○ 0 Worst possible life

Q5.1 On a scale of 1 (Disagree a lot) to 5 (Agree a lot), please read the below statements and select the answer you feel is most appropriate to you:

	Disagree a lot (1)	Disagree a little	Neither Agree nor Disagree	Agree a little	Agree a lot (5)
I can be physically active during my free time on most days.	○	○	○	○	○
I can ask my parent or other adult to do physically active things with me.	○	○	○	○	○
I can be physically active during my free time on most days even if I could watch TV or play video games instead.	○	○	○	○	○
I can be physically active during my free time on most days even if it is very hot or cold outside.	○	○	○	○	○
I can ask my best friend to be physically active with me during my free time on most days.	○	○	○	○	○
I can be physically active during my free time on most days even if I have to stay at home.	○	○	○	○	○
I have the coordination I need to be physically active during my free time on most days.	○	○	○	○	○
I can still be physically active during my free time on most days no matter how busy my day is.	○	○	○	○	○

Q5.2 When I am active...

	Disagree a lot (1)	Disagree a little	Neither Agree nor Disagree	Agree a little	Agree a lot (5)
... I enjoy it	○	○	○	○	○
... I feel bored.	○	○	○	○	○
... I dislike it.	○	○	○	○	○
... I find it pleasurable	○	○	○	○	○
... it's no fun at all.	○	○	○	○	○
... it gives me energy	○	○	○	○	○
... it makes me depressed	○	○	○	○	○
... it's very pleasant	○	○	○	○	○
...my body feels good	○	○	○	○	○
... I get something out of it	○	○	○	○	○
... it's very exciting	○	○	○	○	○
... it frustrates me.	○	○	○	○	○
... it's not at all interesting.	○	○	○	○	○
... it gives me a strong feeling of success	○	○	○	○	○
... it feels good	○	○	○	○	○
... I feel as though I would rather be doing something else.	○	○	○	○	○

Q6.1 On a scale of 1 (dislike very much) to 5 (like very much), please tell us how much you liked the following aspects of the Online Girls Active Project

	Dislike very much (1)	Dislike somewhat	Neither like nor dislike	Like somewhat	Like very much (5)
Organisation	○	○	○	○	○
Length of delivery (duration of classes)	○	○	○	○	○
Date(s) of delivery	○	○	○	○	○
Start and end time	○	○	○	○	○
It being online	○	○	○	○	○
Variety of activities each week	○	○	○	○	○
Information provided	○	○	○	○	○
Delivery of activity: Project leaders (Transition Years) delivering the classes	○	○	○	○	○
Overall satisfaction	○	○	○	○	○

Q7.1 On a scale of 1 (not at all) to 5 (extremely), please tell us how much the following aspects of the Online Girls Active Project encouraged or motivated you to participate:

	**Not at all (1)**	**Slightly (2)**	**Moderately (3)**	**Very (4)**	**Extremely (5)**
The Girls Active Project Certificate	○	○	○	○	○
Prizes (vouchers, etc.)	○	○	○	○	○
My friends	○	○	○	○	○
My family	○	○	○	○	○
Being with others	○	○	○	○	○
Meeting new people	○	○	○	○	○
Wanting to be physically fit	○	○	○	○	○
Improving my health and well-being	○	○	○	○	○
Improving my cardiovascular fitness	○	○	○	○	○
Learning new skills	○	○	○	○	○
Challenging myself	○	○	○	○	○

Q8.1 Would you like for this online Girls Active Project to stay in the school?

Yes	No	Unsure

Q8.2 Would you continue to participate in the online Girls Active Project if it stayed in the school permanently?

Yes	No	Unsure

Q9. Do you have any other comments you would like to share about the Girls Active Project?

Please feel free to make any additional observations here:

____

____

____

____

Thank you for taking the time to complete this questionnaire.
Your response has been recorded.

We hope you enjoy the Girls Active Project!

Intervention Recipients: Post-Questionnaire

Remember:

1. There are no right or wrong answers - this is not a test.
2. Please answer all of the questions as honestly and accurately as you can - this is very important.

The information gathered will be kept strictly confidential. No identifying information about you will ever be published or shared.

You can play 2 or 3 of your favourite songs, drink a cup of tea and complete the questionnaire (it will take approximately 10 minutes to complete).

Today's Date (dd/mm/yyyy):

____

Q1.1 Name:
(this will be replaced with an I.D. Code)

____

You were provided with instructions and asked to measure your height (in centimetres) and weight (in kilograms) at home.
Please note your measurements below:

Q2.1 Height (cm): _______________ *(leave blank if you'd rather not say)*

Q2.2 Weight (kg) to the nearest .1kg: _______________ *(leave blank if you'd rather not say)*

Read the following statements before answering the two questions below.
Physical activity is any bodily movement.
Physical activity includes: Exercises - Weight training, aerobics, jogging, dancing, etc. Sports - Hurling, football, athletics, swimming, etc. General - Brisk walking, washing the car, walking or cycling to school, etc. It can be done at different levels of effort:

<u>Moderate Effort</u> makes your heart rate and breathing rate faster than normal. You may also sweat a little. Brisk walking and jogging are good examples.
<u>Vigorous Effort</u> makes your heart rate much faster, and you have to breathe deeper and faster than normal. You will probably sweat. Playing football or tennis are good examples.

Please try to think carefully and be as accurate as possible with your answers.
For these next two questions, add up all the time you spend in physical activity each day. Only include activities of either MODERATE or VIGOROUS effort (as described above).

Q3.1 Over the past 7 days, on how many days were you physically active for a total of at least 60 minutes per day? <u>Please tick one number.</u>

0 days	1 day	2 days	3 days	4 days	5 days	6 days	7 days
○	○	○	○	○	○	○	○

Q3.2. Over a typical or usual week, on how many days are you physically active for a total of at least 60 minutes per day? Only include activities of either MODERATE or VIGOROUS effort. <u>Please tick one number.</u>

0 days	1 day	2 days	3 days	4 days	5 days	6 days	7 days
○	○	○	○	○	○	○	○

The following questions will ask you about your health and well-being.

Q4.1 Would you say your health is...?

- ◯ Excellent
- ◯ Good
- ◯ Fair
- ◯ Poor

Q4.2 Here is a ladder. The top of the ladder "10" is the best possible life for you and the bottom "0" is the worst possible life for you.
In general, where on the ladder do you feel you stand at the moment?

- ◯ 10 Best possible life
- ◯ 9
- ◯ 8
- ◯ 7
- ◯ 6
- ◯ 5
- ◯ 4
- ◯ 3
- ◯ 2
- ◯ 1
- ◯ 0 Worst possible life

Q5.1 On a scale of 1 (Disagree a lot) to 5 (Agree a lot), please read the below statements and select the answer you feel is most appropriate to you:

	Disagree a lot (1)	Disagree a little	Neither Agree nor Disagree	Agree a little	Agree a lot (5)
I can be physically active during my free time on most days.	○	○	○	○	○
I can ask my parent or other adult to do physically active things with me.	○	○	○	○	○
I can be physically active during my free time on most days even if I could watch TV or play video games instead.	○	○	○	○	○
I can be physically active during my free time on most days even if it is very hot or cold outside.	○	○	○	○	○
I can ask my best friend to be physically active with me during my free time on most days.	○	○	○	○	○
I can be physically active during my free time on most days even if I have to stay at home.	○	○	○	○	○
I have the coordination I need to be physically active during my free time on most days.	○	○	○	○	○
I can still be physically active during my free time on most days no matter how busy my day is.	○	○	○	○	○

Q5.2 When I am active...

	Disagree a lot (1)	Disagree a little	Neither Agree nor Disagree	Agree a little	Agree a lot (5)
... I enjoy it	○	○	○	○	○
... I feel bored.	○	○	○	○	○
... I dislike it.	○	○	○	○	○
... I find it pleasurable	○	○	○	○	○
... it's no fun at all.	○	○	○	○	○
... it gives me energy	○	○	○	○	○
... it makes me depressed	○	○	○	○	○
... it's very pleasant	○	○	○	○	○
...my body feels good	○	○	○	○	○
... I get something out of it	○	○	○	○	○
... it's very exciting	○	○	○	○	○
... it frustrates me.	○	○	○	○	○
... it's not at all interesting.	○	○	○	○	○
... it gives me a strong feeling of success	○	○	○	○	○
... it feels good	○	○	○	○	○
... I feel as though I would rather be doing something else.	○	○	○	○	○

Q6.1 On a scale of 1 (dislike very much) to 5 (like very much), please tell us how much you liked the following aspects of the in-person Girls Active Project

	**Dislike very much (1)**	**Dislike somewhat**	**Neither like nor dislike**	**Like somewhat**	**Like very much (5)**
Organisation	○	○	○	○	○
Length of delivery (duration of classes)	○	○	○	○	○
Date(s) of delivery	○	○	○	○	○
Start and end time	○	○	○	○	○
It being in-person	○	○	○	○	○
Variety of activities each week	○	○	○	○	○
Information provided	○	○	○	○	○
Delivery of activity: Project leaders (Transition Years) delivering the classes	○	○	○	○	○
Overall satisfaction	○	○	○	○	○

Q6.2 Did you prefer participating in the Girls Active Project online or in-person?

**Online**	**In-Person**	**Unsure**	**Liked both equally**

Q7.1 On a scale of 1 (not at all) to 5 (extremely), please tell us how much the following aspects of the in-person Girls Active Project encouraged or motivated you to participate:

	Not at all (1)	Slightly (2)	Moderately (3)	Very (4)	Extremely (5)
The Girls Active Project Certificate	○	○	○	○	○
Prizes (vouchers, etc.)	○	○	○	○	○
My friends	○	○	○	○	○
My family	○	○	○	○	○
Being with others	○	○	○	○	○
Meeting new people	○	○	○	○	○
Wanting to be physically fit	○	○	○	○	○
Improving my health and well-being	○	○	○	○	○
Improving my cardiovascular fitness	○	○	○	○	○
Learning new skills	○	○	○	○	○
Challenging myself	○	○	○	○	○

Q8.1 Would you like for the in-person Girls Active Project to stay in the school?

Yes	No	Unsure

Q8.2 Would you continue to participate in the in-person Girls Active Project if it stayed in the school permanently?

Yes	No	Unsure

Q8.3 Is there anything the school can do to encourage or support you to take part in the Girls Active Project?

____

____

____

____

Q9.1 List the top three things you liked most about the Girls Active Project (online and/or in-person):

_____1.____

_____2.____

_____3.____

Q9.2 List the top three things you liked least (or things that need improving) about the Girls Active Project (online and/or in-person):

_____1.____

_____2.____

_____3.____

Q10.1 Were there any barriers to you maintaining involvement in the Girls Active Project?

Yes	No	Unsure

Q10.2 If you answered 'yes' to the above, what were the barriers to you maintaining involvement?

____

____

____

Q11.1 Did you understand the relevance of the Girls Active Project?

Yes	No	Unsure

Q11.2 Do you think the current pandemic (COVID-19) impacted your participation in the Girls Active Project?

Yes	No	Unsure

Q11.3 Please explain how the current pandemic did/did not impact your participation in the Girls Active Project:

____

____

____

Q11.4 Do you think the questionnaires and focus group(s) with the researcher (Sara) as part of this study were acceptable?

Yes	No	Unsure

Q12.1 Do you have any other comments you would like to share about the Girls Active Project?

Please feel free to make any additional comments here:

____

____

____

Thank you for taking the time to complete this questionnaire.
We hope you enjoyed the Girls Active Project!

Mid-intervention: Focus Group Topic Guide
Intervention Recipients

Acceptability – Satisfaction

1. Were you happy with the GAP online?
2. Were you engaged with the GAP online?
3. Were there any exercise classes online that you particularly liked/disliked?
4. Did you like the dates, times, length/duration, variety (exercises) of the classes?
5. Did you like that the TYs (Project Leaders) delivered the classes?
6. Is there anything (more) the school could do to support or encourage you to participate in the GAP?

Retention

7. What aspects of the GAP encouraged/motivated you to participate? (i.e., what motivated you to participate in the GAP?)

   E.g., seeing friends, improve health, learn new skills, win prizes

Implementation + Context

8. Could you understand the relevance/purpose of the GAP?
9. What helped and/or hindered you attending online? [barriers/facilitators]

Compatibility – perceived sustainability

10. Will you continue to participate in the GAP when it's delivered in-person?
11. Would you continue if it were delivered online?

12. Would you continue to participate in the GAP if it stayed permanently in the school?

13. How can we get more second years to participate?

Additional

14. Any further comments you'd like to make about the Girls Active Project – Online and/or in-person?

Post-intervention: Focus Group Topic Guide

Intervention Recipients

Retention

1. Why did you participate in the GAP? What aspects of the GAP encouraged/motivated you to participate? (i.e., what motivated you to participate in the GAP after school?) e.g., seeing friends, improve health, learn new skills, win prizes

Acceptability – Satisfaction

2. Overall, what did you like most about the GAP? And what did you like least about the GAP? Did you prefer it online or face-to-face?
3. Were you happy with the GAP at school (in-person delivery)?
4. Did you like the dates, times, length/duration, variety (exercises) of the classes?
5. Did you like that the TYs (Project Leaders) delivered the classes?
6. Were the questionnaires and focus groups as part of this study okay?
7. Is there anything (more) the school could do to support or encourage you to participate in the GAP?

Implementation + Context

8. Could you understand the relevance/purpose of the GAP?

9. What factors helped or hindered you to attend the GAP after school? <u>And/or maintain involvement?</u>
    a. Do you think the current pandemic (COVID-19) impacted (positively and/or negatively to) your participation in the GAP? (and if so, how?)

Compatibility – perceived sustainability

10. Moving forward, would you like for the in-person delivered GAP to remain as an option in the school?
11. Would you continue to participate in the GAP if it stayed permanently in the school?
12. Would you recommend your classmate/friends to participate in the GAP?
13. Do you think it could work as a whole-school programme? i.e., all students (year one to six) would be invited to join and TYs deliver the classes. Why/why not?

Additional

14. Any further comments you'd like to make about the Girls Active Project – Online and/or in-person?

Health Research Board | Girls Active Project | DCU

Mid-intervention: Focus Group Topic Guide
Project Leaders

Role: Project Leaders delivered the intervention (intervention providers)

Acceptability – Satisfaction

1. Did you enjoy being a project leader? Why/why not?
2. How happy were you with the GAP online?
3. In your opinion, how happy were the second years [intervention recipients] with the GAP online?
4. In your opinion, were the second years [intervention recipients] engaged in the GAP online? (Even with their cameras off)
5. Did you like the dates, times, length/duration, variety (content) of the classes?

Implementation + Context

6. Was the intervention implemented as planned?
7. What factors helped and/or hindered you to deliver the GAP online? [barriers and/or facilitators that affected GAP online implementation]
8. Was there (and if so, what) strategies/structures (were) built by the school to support the GAP?
9. Is there anything more the school can do to support or encourage you (and other students) to participate in the GAP?

Compatibility – sustainability

10. Do you think (and if so, to what extent) would the online GAP be sustainable/used long-term in the school?
11. Will you continue to deliver the GAP when it's in-person?
12. How can we get more second years (intervention recipients) to participate?

Additional

13. Any further comments you'd like to make about the Girls Active Project intervention?

HRB Health Research Board

Girls Active Project

DCU

Post-intervention: Focus Group Topic Guide
Project Leaders

Role: Project Leaders delivered the intervention (intervention providers)

Acceptability – Satisfaction

1. Did you enjoy being a project leader? Why/why not?
2. Did you enjoy working as part of a team? Do you think you all worked well as a team?
3. Were you happy with the GAP?
4. In your opinion, were the second years (intervention recipients) engaged and/or happy with the GAP?
5. Did you like the dates, times, length/duration, variety (content) of the classes?
6. How did you find the focus groups and feedback questionnaire as part of this study?

Implementation + Context

7. As a project leader, how was it completing the weekly logbooks?
8. Were there any barriers to you being a Project Leader? If so, what were they?
9. Could you understand the relevance/purpose of the GAP?
10. Do you think the GAP was implemented as planned?
11. What factors helped and/or hindered delivering the GAP?
    - Do you think the current pandemic (COVID-19) impacted (positively and/or negatively) participation in the GAP? (and if so, how?)
12. Were there strategies/structures built by the school to support the GAP? (and if so, what were they?)

13. Is there anything more the school can do to support or encourage you (and other students) to participate in the GAP?

Compatibility + Retention

14. Why did you become a project leader? [What aspects of the GAP encouraged/motivated you to participate?] e.g., seeing friends, improve health, learn new skills, win prizes
15. What did you gain from being a project leader? What are the advantages/disadvantages of being a GAP project Leader? For example, would you recommend other students to become a project leader? And why/why not?
16. Do you think (and if so, to what extent) would the GAP be sustainable/used long-term in the school?
17. Would you continue to participate in the GAP if it stayed permanently in the school?
18. Moving forward, do you think it could work as a whole-school programme? Where all students (year one to six) are invited to join after school and Project Leaders deliver the programme. Why/why not?

Additional

19. Any further comments you'd like to make about the Girls Active Project intervention?

Welcome to the Girls Active Project
Project Leaders Feedback Questionnaire (Post-Intervention)

Remember:
1. There are no right or wrong answers - this is not a test.
2. Please answer all the questions as honestly and accurately as you can - this is very important.

It is anonymous. The information gathered will be kept strictly confidential. No identifying information about you will ever be published or shared. It will take approximately 10-15 minutes to complete.

Today's Date (dd/mm/yyyy):

____

Q1.1 List the top three things you liked most about being a Project Leader in the Girls Active Project:

1. ____
2. ____
3. ____

Q1.2 List the top three things you liked least (or things that need improving) about being a Project Leader in the Girls Active Project:

1. ____
2. ____
3. ____

Q1.3 On a scale of 1 (not at all) to 5 (extremely), please tell us how much the following aspects of the Girls Active Project encouraged or motivated you to be a Leader:

	Not at all (1)	Slightly (2)	Moderately (3)	Very (4)	Extremely (5)
The Girls Active Project Certificate	○	○	○	○	○
Prizes (vouchers, etc.)	○	○	○	○	○
My friends	○	○	○	○	○
My family	○	○	○	○	○
Being with others	○	○	○	○	○
Meeting new people	○	○	○	○	○
Wanting to be physically fit	○	○	○	○	○
Improving my health and well-being	○	○	○	○	○
Improving my cardiovascular fitness	○	○	○	○	○
Develop/Improve leadership skills	○	○	○	○	○
Develop/Improve communication skills	○	○	○	○	○
Develop/Improve teamwork skills	○	○	○	○	○
Challenging myself	○	○	○	○	○

Q1.4 Are there any other aspects of the Girls Active Project that encouraged or motivated you to be a Leader? If so, please write them here:

____

____

____

Q1.5.1 Would you recommend being a Project Leader to other students?

Yes	No	Unsure

Q1.5.2 Please explain, why you would/wouldn't recommend being a Project Leader (i.e., what are the advantages/disadvantages of being a GAP Project Leader?)

____

____

____

____

Q1.6 If you were involved in the Girls Active Project again, what would you change?

____

____

____

____

Q1.7.1 Were there any barriers to you maintaining involvement in the Girls Active Project?

Yes	No	Unsure

Q1.7.2 If you answered 'yes' to the above, what were the barriers to you maintaining involvement?

____

____

____

Health Research Board

Girls Active Project

DCU

Q2.1 On a scale of 1 (dislike very much) to 5 (like very much), please tell us how much you liked the following aspects of the Girls Active Project

	Dislike very much (1)	Dislike somewhat	Neither like nor dislike	Like somewhat	Like very much (5)
Organisation	○	○	○	○	○
Length of delivery (duration of classes)	○	○	○	○	○
Date(s) of delivery	○	○	○	○	○
Start and end time	○	○	○	○	○
Being a leader delivering the classes	○	○	○	○	○
Variety of activities each week	○	○	○	○	○
Completing the weekly logbooks	○	○	○	○	○
Working as part of a team	○	○	○	○	○
Overall satisfaction	○	○	○	○	○

Q2.2 Did you prefer being a Project Leader for the Girls Active Project online or in-person?

Online	In-Person	Unsure	Liked both equally

Q3.1 Did you understand the relevance of the Girls Active Project?

Yes	No	Unsure

Q3.2 Do you think the current pandemic (COVID-19) impacted participation in the GAP?

Yes	No	Unsure

Q3.3 In your opinion, please explain how the current pandemic impacted participation in the GAP:

____

____

____

____

Q4.1 Would you like for the Girls Active Project to stay in the school?

Yes	No	Unsure

Q4.2 Would you continue to participate in the Girls Active Project if it stayed in the school permanently?

Yes	No	Unsure

Q4.3 Is there anything more the school can do to encourage or support you to take part in the Girls Active Project?

____

____

____

____

Q5.1 Do you have any other comments you would like to share about the Girls Active Project?

If so, please feel free to make any additional observations here:

____

____

____

THANK YOU ☺

Parents/Guardians Questionnaire - Online (post-intervention)

Hello and thank you for taking part in this survey.

The following questions will ask you about the Girls Active Project (after school programme Tuesdays, 4pm) at your daughter's school. This is a study being carried out by Dublin City University. We are testing a school-based programme aimed at improving the physical activity levels of teenage girls. We are inviting you to complete this survey to gain insight into your views of the after-school programme. There are no potential risks from involvement. Participation is voluntary and you may withdraw from the survey at any point. The information you provide will be kept strictly confidential and your participation in the study will be private. The information will be stored securely and in a way that protects your identity. If you have any question about the study, please contact Sara McQuinn (087-6121709, sara.mcquinn2@mail.dcu.ie).

If participants have concerns about this study and wish to contact an independent person, please contact: *The Secretary, Dublin City University Research Ethics Committee, c/o Research and Innovation Support, Dublin City University, Dublin 9. Tel 01-7008000 e-mail* **rec@dcu.ie**

There are no right or wrong answers and please be as honest as you can. It will take approximately 10 minutes to complete.

Consent
Please note that your responses to this survey are anonymous and your participation will be kept strictly confidential.
I agree to take part in this survey:

○ Yes

○ No

Q1 Todays Date (dd/mm/yyyy):

Q2 Did you know about the Girls Active Project (after school programme, 4pm Tuesdays) at your daughter's school?

- ◯ Yes (1)
- ◯ No (2)
- ◯ Unsure (3)

Q3 Did you understand the relevance of the Girls Active Project?

- ◯ Yes (1)
- ◯ No (2)
- ◯ Unsure (3)

Q4 Did your daughter take part in the Girls Active Project (after school programme, 4pm Tuesdays)?

- ◯ Yes (1)
- ◯ No (2)

Display This Question:

If Did your daughter take part in the Girls Active Project (after school programme, 4pm Tuesdays)? = Yes

Q4.1.1 If yes, why do you think she participated?

____

____

Display This Question:

If Did your daughter take part in the Girls Active Project (after school programme, 4pm Tuesdays)? = Yes

Q4.1.2 Please read the below statements and in your opinion, on a scale of 1 (disagree a lot) to 5 (agree a lot), select the answer you feel is most appropriate to you:

My daughter...

	Disagree a lot (1)	Disagree a little	Neither agree nor disagree	Agree a little	Agree a lot (5)	Not Applicable
... enjoyed participating n the Girls Active Project	○	○	○	○	○	○
... found the programme fun and social	○	○	○	○	○	○
... joined because she wanted to be with her friends/meet new people	○	○	○	○	○	○
... participated because she wanted to improve her health and fitness	○	○	○	○	○	○
... participated because there were prizes/rewards	○	○	○	○	○	○
... liked that the Transition Year students delivered the exercise classes	○	○	○	○	○	○
... liked the timing, dates, and duration of the exercise classes	○	○	○	○	○	○
... liked that there was a variety of exercise classes	○	○	○	○	○	○
... would continue to participate in the Girls Active Project if it were to remain in the school	○	○	○	○	○	○

Display This Question:

If Did your daughter take part in the Girls Active Project (after school programme, 4pm Tuesdays)? = Yes

Q4.1.3 Are there any other aspects of the Girls Active Project your daughter seemed to like/dislike? If so, please write them here:

____

____

____

Display This Question:

If Did your daughter take part in the Girls Active Project (after school programme, 4pm Tuesdays)? = No

Q4.2 If not, why do you think she did not participate?

____

____

____

Q5 Would you like the Girls Active Project (after-school programme) to remain as an option to students at the school?

- ◯ Yes (1)
- ◯ No (2)
- ◯ Unsure (3)

Q6 Please indicate if there is anything that the school can do to make it easier for you as a parent to encourage or support your daughter to take part in the Girls Active Project?

____

Q7 Are there any comments or recommendations about the Girls Active Project you'd like to add? If so, please write them here:

____

____

____

End of Block: Evaluation

Thank you very much for completing the survey. By clicking the 'Next' tab your response will be recorded and you will be re-directed to another form.

Re-directed to another Qualtrics form:

GAP: Parents' Consent to Phone Interview

Thank you.

If you are willing to take part in a short (5-10 minute) recorded phone call interview with the lead researcher, Sara McQuinn, please leave your details below. An audio recorder will be used to record the phone call. The recording will be deleted after transcription. No identifying information will be on the transcription.

Participation will be kept strictly confidential; your information will be stored securely and in a way that protects your identity. By leaving your details below, you agree to take part in a recorded phone call interview.

Q1 Parent/Guardian Name:

____

Q2 Telephone Number:

____

Thank You Thank you for completing the form. We will be in touch shortly.

Post-intervention: Semi-structured interview questions
Parent/Guardian

Implementation + Context

1. Can you remember how you heard/found out about the Girls Active Project (GAP)?
2. Could you understand the relevance (purpose) of GAP?
3. In your opinion, were there any factors that helped or hindered your daughter participating in the GAP? (Online and/or in-person)
   [barriers and/or facilitators that affected GAP implementation]
4. To what extent did your daughter get involved/engage with the GAP?

Acceptability - Satisfaction

5. Was she happy with the GAP? Did your daughter enjoy being involved in the Girls Active Project? Why/why not?
6. What did she like/dislike about the GAP?
7. Did she prefer it online or in-person (if applicable)?
8. Did you think the timing (after-school) and duration of classes (45 mins) were okay? Delivery (peer-led) and content (variety of activities) delivered were acceptable?
9. Is there anything the school can do to make it easier for you as a parent to encourage or support your daughter to take part in the GAP?

Compatibility – Sustainability

10. Do you think the GAP could be used long-term in the school? (Do you think there is potential?)
11. Would you like the GAP to remain as an option at the school?
12. In your opinion, what does the GAP have to offer that the other school sports teams and clubs don't?
13. Do you think your daughter would continue to be involved in/support the GAP if it stayed permanently in the school?

Additional Q

14. Any further comments, observations, recommendations you'd like to make about the GAP?

Thank you so much for your time

Post-intervention: Semi-structured interview questions

School Staff Members

Implementation + context

1. To what extent did you/your school/the students get involved/engage with the Girls Active Project (GAP)?
2. Could you understand the relevance of GAP?
3. In your opinion, what factors helped or hindered the GAP being implemented? (Online and in-person) [barriers and/or facilitators that affected GAP implementation]
    a. Do you think the current pandemic (COVID-19) impacted (positively and/or negatively) participation in the GAP? (and if so, how?)
4. What strategies & structures were built by the school to support the GAP?

Acceptability – Satisfaction

5. Did you/your school enjoy being involved in the Girls Active Project? Why/why not? [engagement/responsiveness]
6. What did you like/dislike about the GAP?
7. Were you happy with the GAP?
8. In your opinion, were the students (second years [intervention recipients] and Project Leaders [intervention providers]) engaged and/or happy with the GAP?
9. Did you like the classes - dates, times, length/duration, content variety, (peer-led) delivery?
10. Do you think the research and/or extra workload as part of this study was acceptable? (i.e., communication with research team, interviews and/or focus groups, sharing info. on social media, contacting parents, planning, emailing students, arranging zoom classes, etc.)?

Compatibility – sustainability

11. Do you think the GAP could be sustainable/used long-term in the school? (Do you think there is potential? And if so, to what extent?)
12. In your opinion, what does the GAP have to offer that the other school sports teams and clubs don't?
13. Would you continue to be involved in/support the GAP if it stayed permanently in the school?
14. Moving forward, do you think it could work as a whole-school programme? Where all students (year one to six) are invited to join after school and Project Leaders deliver the programme. Why/why not?
15. If you were to be involved in the GAP again, is there anything you would you do differently? (and if so, what?)

Additional Q

16. Any further comments you'd like to make about the GAP?
