# Supplementary material for: Feasibility of a peer-led, after-school physical activity intervention for disadvantaged adolescent females during the COVID-19 pandemic: results from the Girls Active Project (GAP)
Source: Pilot Feasibility Stud. 2022 Aug 30;8:194. doi: 10.1186/s40814-022-01149-2 (PMC9425823; doi:10.1186/s40814-022-01149-2)
Supplement: Supplementary file 4 — Additional file 4: Supplementary file 4. GAP Materials [file 40814_2022_1149_MOESM4_ESM.pdf]

Girls Active Project Plan

Class	Date/Time	Project Leaders Delivering Class	Exercise/Activity
1			
2			
3			
4			
5			
6			
7			
8			

Exercise Classes: Suggested by Project Leaders

- Dance
- Circuits
- Boxercise
- Football (Soccer)
- Yoga
- GAA Football
- Zumba
- Relay-races
- Hip-hop
- Hurdles
- Basketball
- Boxing
- ‘Just Dance’ videos
- H.I.I.T.

School Name

Certificate of Achievement

Presented to Project Leader

In recognition of your time and
dedication to the
Girls Active Project

Project Manager

Date

Girls Active Project

HRB Health Research Board

SPHeRE Structured Population and Health-services Research Education

DCU

School Name

Certificate of Award

This acknowledges that

____

has successfully participated in the
Girls Active Project

____
Project Manager

____
Date

Girls Active Project

Health Research Board

SPHeRE
Structured Population and Health-services Research Education

DCU
